# Using formative research to develop the healthy eating component of the CHANGE! school-based curriculum intervention

**DOI:** 10.1186/1471-2458-12-710

**Published:** 2012-08-29

**Authors:** Lynne M Boddy, Zoe R Knowles, Ian G Davies, Genevieve L Warburton, Kelly A Mackintosh, Laura Houghton, Stuart J Fairclough

**Affiliations:** 1 The Research Institute for Sport and Exercise Sciences, Liverpool John Moores University, Tom Reilly Building, Byrom Street, Liverpool L3 3AF, UK; 2 The Faculty of Education, Community and Leisure, Liverpool John Moores University, IM Marsh Campus, Barkhill Road, Liverpool, L17 6BD, UK

**Keywords:** Nutrition, Childhood obesity, Pen-profiles, Health, Schools

## Abstract

**Background:**

Childhood obesity is a significant public health concern. Many intervention studies have attempted to combat childhood obesity, often in the absence of formative or preparatory work. This study describes the healthy eating component of the formative phase of the Children’s Health Activity and Nutrition: Get Educated! (CHANGE!) project. The aim of the present study was to gather qualitative focus group and interview data regarding healthy eating particularly in relation to enabling and influencing factors, barriers and knowledge in children and adults (parents and teachers) from schools within the CHANGE! programme to provide population-specific evidence to inform the subsequent intervention design.

**Methods:**

Semi-structured focus group interviews were conducted with children, parents and teachers across 11 primary schools in the Wigan borough of North West England. Sixty children (N = 24 boys), 33 parents (N = 4 male) and 10 teachers (N = 4 male) participated in the study. Interview questions were structured around the PRECEDE phases of the PRECEDE-PROCEED model. Interviews were transcribed verbatim and analysed using the pen-profiling technique.

**Results:**

The pen-profiles revealed that children’s knowledge of healthy eating was generally good, specifically many children were aware that fruit and vegetable consumption was ‘healthy’ (N = 46). Adults’ knowledge was also good, including restricting fatty foods, promoting fruit and vegetable intake, and maintaining a balanced diet. The important role parents play in children’s eating behaviours and food intake was evident. The emerging themes relating to barriers to healthy eating showed that external drivers such as advertising, the preferred sensory experience of “unhealthy” foods, and food being used as a reward may play a role in preventing healthy eating.

**Conclusions:**

Data suggest that; knowledge related to diet composition was not a barrier per se to healthy eating, and education showing how to translate knowledge into behavior or action is required. The key themes that emerged through the focus groups and pen-profiling data analysis technique will be used to inform and tailor the healthy eating component of the CHANGE! intervention study.

**Trial registration:**

Current Controlled Trials ISRCTN03863885

## Background

The increased prevalence of childhood obesity and overweight has been widely documented [1], and the negative health implications of excessive adiposity are well established [2]. Despite evidence suggesting that the prevalence of childhood obesity has reached a plateau, a large proportion of children remain overweight or obese and prevalence shows no sign of reducing [1]. Children’s food intake and eating behaviours in conjunction with insufficient levels of physical activity have been cited as key factors in the obesity ‘epidemic’, with food accounting for the ‘energy in’ component of the energy balance [3]. In particular, readily available energy-dense foods, and energy containing beverages have been implicated as ‘causes’ of excessive adiposity in children and young people, despite evidence to suggest energy intake has not increased substantially in recent decades years [4]. However, in addition to maintaining an appropriate energy balance, there are a number of other benefits to adopting a healthy diet in youth. In particular, intakes of fruit and vegetables (FV) are linked to a reduced risk of a number of conditions including various cancers and cardiometabolic disease [5,6]. As many disease processes begin in youth [7], and obesity tracks from childhood through to adulthood [8], it is important that healthful behaviours are adopted at a young age. A number of intervention studies have attempted to promote healthy body size through improving the eating habits of children, often with limited levels of success, and recent systematic reviews suggest multi-component studies that address both sides of the energy equation (i.e. physical activity and healthy eating) within interventions are the most effective [9].

The National Institute of Clinical Excellence guidelines highlight a number of important factors for behavior change [10]. For example, when designing an intervention it is of importance to understand the circumstances, needs, and assets of the target population, as well as involve the target population within the development of the intervention itself. By facilitating the target population to assess their own needs and barriers, compliance to a tailored programme is more likely to be both successful and sustainable for the participants [11,12]. Furthermore, it is important to incorporate an appropriate theoretical model that can develop and augment the strengths and assets of the target group within intervention design [10]. In the context of these guidelines formative work should be viewed as a critical step within intervention design. This paper describes formative work undertaken to inform the design of one component of the Children’s Health, Activity and Nutrition: Get Educated! (CHANGE!), school-based curriculum intervention study. Mackintosh et al. [13] have previously detailed similar formative work to inform the design of a physical activity intervention within CHANGE!. Views elicited on physical activity were consistent across both parents and children and it was noted that families play a potentially powerful and important role in promoting health-enhancing behaviours. The aim of the present study was to gather qualitative focus group and interview data regarding healthy eating particularly in relation to enabling and influencing factors, barriers and knowledge in both children and adults (parents and teachers) from schools within the CHANGE! programme to provide important population-specific evidence to inform intervention design.

## Methods

The methods for the CHANGE! formative work have been described elsewhere [13]. Briefly, fourteen schools across the Wigan Borough, North-West England, were invited to take part in the formative phase of the study. Eleven schools agreed to participate. The schools were clustered within administrative areas known as Neighbourhood Management Areas and stratified by free school meal entitlement (as a proxy for socio-economic status). Two schools from each NMA were recruited, one classified as high and one classified as low SES. An additional high SES school was included from one area due to school withdrawal and re-enrolment in the study. All participants’ ethnicity classification was ‘white British’, which is representative of the Wigan Borough population.

After gaining institutional ethical approvals from Liverpool John Moores University Research Ethics Committee, informed parental consent and participant assent, 203 Year 5 (9–10.9 yrs old) children were eligible to take part in the study. For the formative component of CHANGE! a random sub-sample of children, stratified by sex, were selected to take part in focus groups using a random number generator. Children’s parents and class teachers were also invited to take part in group interviews and individual interviews, respectively. Sixty children (N = 24 boys, 36 girls), 33 parents (N = 4 male, 29 female) and 10 teachers (N = 4 male, 6 female) participated in the study.

### Procedures

The procedures for data collection have been described in detail elsewhere [13]. Thirteen semi-structured group interviews were conducted by one researcher, each involving 3–5 child participants. A rationale for this methodology with children has been provided previously (see Mackintosh et al., 2011, [13]). Nine group interviews were conducted with parents (3–8 participants per interview). Seven individual interviews and one small group interview (2 participants) were conducted with teachers. All interviews were constructed using the PRECEDE (predisposing, reinforcing and enabling constructs) component of the PRECEDE-PROCEDE model as a guide for questions and topics to cover [14]. Questions were tailored to suit the age and type of participant and addressed beliefs, knowledge, attitudes and barriers towards healthy eating demonstrating aspects of face validity. Sample interview questions can be viewed in Table 1. For the group interviews with child participants prompts were used to accommodate differing levels of comprehension, competence and attention spans [13,15]. Group and individual interviews were conducted on school sites in an area which allowed the group to be overlooked from a distance but not overheard, and lasted 30–45 minutes (mean = 35.2 minutes). All interviews were recorded using a digital recorder and were transcribed verbatim. Thirty group/individual interviews were conducted which equated to 426 pages of raw transcription data (228, 122, and 76 pages for children, parents and teachers respectively) incorporating physical activity and nutrition topics. 

**Table 1:** Healthy eating focus group interview topics and examples

**Interview**	**Examples**
Children	‘What does it feel like when you feel hungry?’
	‘What things make you want to eat?’
Adults	‘How do you see the role of eating well in being healthy?’
	‘How are your children’s eating habits affected by emotions?’

### Data analysis

This study adopted a pen profile approach to analyse data. Much debate surrounds the most appropriate method of analysing qualitative data, with approaches ranging from manual tagging through to the use of specialist qualitative analysis software packages. However none of these approaches have impacted upon study validity. Research in sport social science and physical activity (a complementary area) has adopted the pen-profiling technique [16,17], [13]. Pen profiles are an appropriate method for representing analysis outcomes using diagrams of key and emergent themes. The pen-profiling technique used with the CHANGE! formative work has been described previously [13]. Briefly, pen profiles were constructed from the transcripts using a manual approach. Frequency count and example verbatim quotes were added to the diagrams to expand the pen profiles and provide context. One researcher, who was independent to the project delivery team, analysed the transcripts and presented the findings to the wider research team by means of co-operative triangulation. The research team cross-examined the data in reverse from pen profiles back to the transcripts. This process allowed authors to offer alternative interpretations of the data, and was repeated until a consensus had been reached.

## Results

### Pen profiles

A deductive approach was used to analyse data, using the PRECEDE component of the PRECEDE-PROCEDE model as a thematic framework. Emergent themes were explored further using an inductive process. Data are presented separately for children and parents and personal demographic variables or factors were explored throughout analysis rather than presented separately.

### Knowledge of healthy foods/a balanced diet

**Figure 1:**
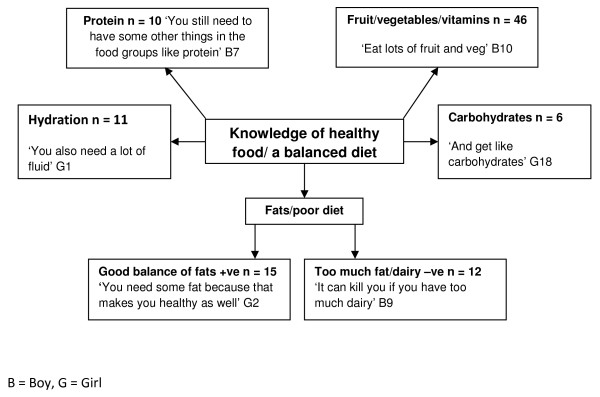
Children’s knowledge of healthy food/a balanced diet.

**Figure 2:**
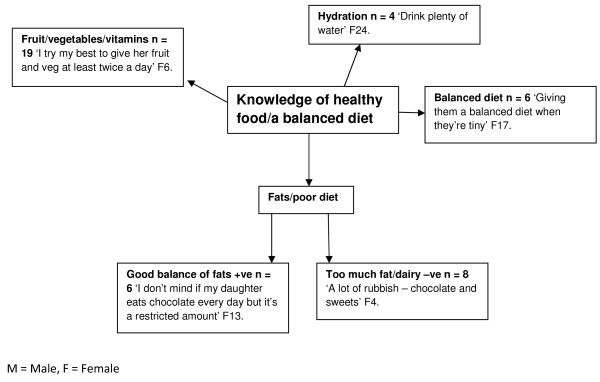
Adult’s knowledge of healthy food/a balanced diet.

Children’s knowledge of healthy eating was generally good (Figure 1). In particular many children were aware that fruit and vegetable consumption was ‘healthy’ (N = 46). In addition, several children had an awareness of food groups (protein N = 10, carbohydrates N = 6, hydration N = 11), and the negative relationship between excessive fat consumption and health (good balance N = 15, too much N =12). Adults’ knowledge was also good, again including promoting fruit and vegetable intake, restricting fatty foods and maintaining a balanced diet (Figure 2).

### Influences to healthy eating

**Figure 3:**
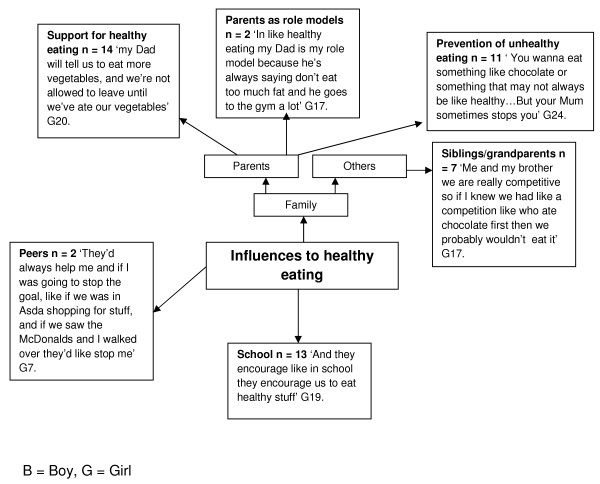
Influences to healthy eating in children.

For children, parents emerged as key influencing factors for healthy eating (Figure 3). Fourteen participants identified parents in providing support for healthy eating, for example:

"‘my dad will tell us to eat more vegetables, and we’re not allowed to leave (the table) until we’ve ate our vegetables’"

Furthermore, children identified parents’ role in preventing unhealthy eating (N = 11), and some children identified parents as role models with regards to healthy eating (N = 2), for example:

"‘in like healthy eating my Dad is my role model because he’s always saying don’t eat too much fat and he goes to the gym a lot’."

Other family members were identified as influential agents healthy eating, including siblings and grandparents (N = 7). Two participants mentioned peers as influential which is an interesting emerging theme in relatively young participants, for example:

"‘They’d (peers) always help me and if I was going to stop the goal, like if we was in Asda shopping for stuff, and if we saw the McDonald’s and I walked over they’d like stop me’"

For children, School was a key influential factor (N = 13). Particularly in encouraging children to eat healthily, for example:

"‘Erm first of all you have veg erm then you pick like a meat or something that's like your main, then you have like erm don't know… Well you have veg then meat then rice maybe. And they encourage like in school they encourage us to eat healthy stuff but they don't just have healthy stuff because like most children don't just like eating loads of healthy stuff so they have a variety.’"

**Figure 4:**
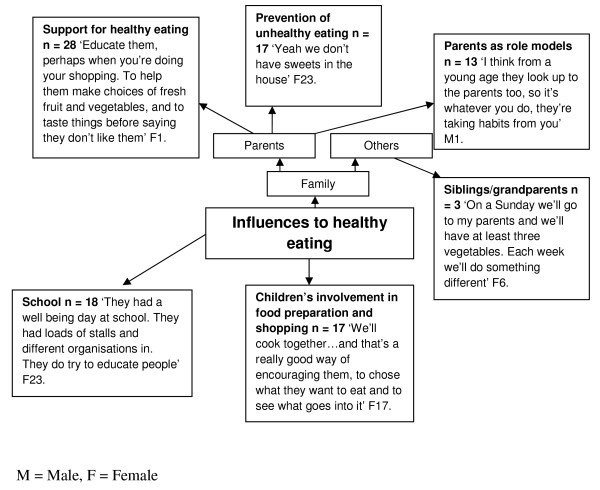
Influences to health eating: adults’ perceptions.

The key emergent themes identified by adults were the role of parents in supporting healthy eating (N = 28), preventing unhealthy eating (N = 17), and parental role-modeling (N = 13) (Figure 4). Other themes emerging from the adult’s interviews were the role of the school in promoting healthy eating (N = 18) for example;

"‘they had a well-being day at school. They had loads of stalls and organizations in. They do try to educate people’"

Adults also identified the role of other family members, in particular siblings and grandparents in reinforcing healthy eating (N = 3). An interesting theme emerging from the adults’ interviews was the importance of children’s involvement in the preparation and purchasing of foods, for example:

"‘We’ll cook together….and that’s a really good way of encouraging them to chose what they want to eat and to see what goes into it’"

### Barriers to healthy eating

**Figure 5:**
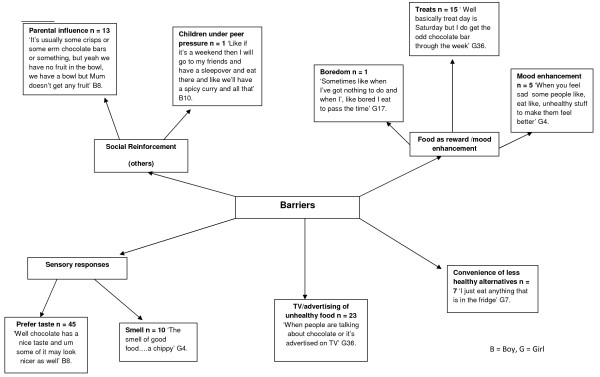
Children’s barriers to healthy eating.

A variety of barriers to healthy eating were identified by children (Figure 5). A major theme emerging from the data were the sensory influences of ‘unhealthy’ foods, for example children preferred the taste (N = 45) and smell (N = 10) of ‘unhealthy’ foods such as:

"‘The smell of good food….a chippy’"

Another barrier identified by many children (N = 23) was the influence of advertising unhealthy foods, including television advertisements

‘When people are talking about chocolate or it’s advertised on TV’

Convenience was an issue raised by seven children, for example:

‘I just eat anything that is in the fridge’

Social reinforcement emerged as a barrier to healthy eating for a number of children, particularly the influence of parents in being responsible for purchasing foods. Food as a reward was cited as a barrier to healthy eating, in particular treats on weekends or for good behavior. The mood enhancing properties of ‘unhealthy’ foods were also discussed by the children.

**Figure 6:**
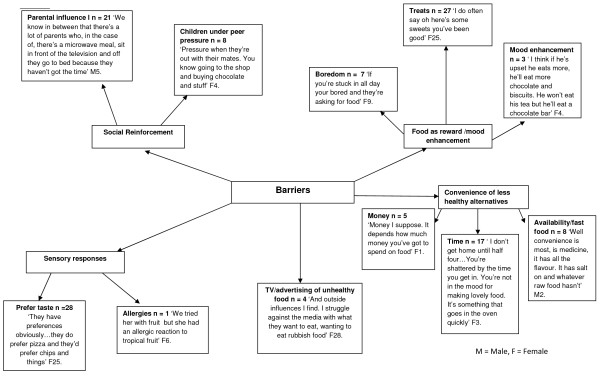
Adults’ perceptions of barriers to healthy eating for children.

For adults, similar themes emerged (Figure 6), for example 28 adults discussed sensory responses to eating ‘unhealthy’ foods:

‘They have preferences obviously……they do prefer pizza and they’d prefer chips and things’

The convenience of less healthy foods emerged as an important theme through the adult interviews, in particular the costs associated with ‘healthy’ food, the time it takes to prepare foods, and that convenience foods are readily available. Food as a reward or mood enhancer was discussed by many of the adults, in particular for rewarding good behavior (N =27).

## Discussion

The aim of the present study was to gather qualitative data regarding healthy eating particularly in relation to enabling and influencing factors, barriers and knowledge in both children and adults (parents and teachers) from schools within the CHANGE! programme to provide important population-specific evidence to inform intervention design. The results provide important information to inform the design of the CHANGE! intervention, and build upon the limited body of literature that has utilized the pen-profiling method of data-analysis. The advantages of the pen-profiling technique, i.e. the capacity to comprehensively review a large data-set aligned with a well accepted theoretical model, whilst removing the likelihood of data being skewed by dominating interview participants, have been documented elsewhere [13]. In the present study pen-profiling has again presented the results of this study in a simple, accessible, yet informative manner though unlike previous research using both adult and child data and a range of group interview sizes (3–5 children, 2–8 adults).

The study findings indicate that children and adults had a generally sound knowledge of the constituents of a balanced diet with high awareness of the importance of fruit and vegetable (FV). These data suggest that knowledge related to diet composition was not a barrier per se to healthy eating within the population group studied but more specific knowledge especially in terms of dairy foods may be required. Previous research evidence is aligned with children’s awareness of healthy food but children’s intake of FV is still below the World Health Organization (WHO) target of 400 g per day [18]. Keyte et al. [19] showed a median of 2 portions of FV intake with schools engaged with the UK Primary School National Healthy Schools Programme compared to only 1 portion per day for other schools. This indicates that whilst health campaigns can improve intake further work is needed to reach WHO and national recommendations. With respect to the specificity of the health benefits of FV, children’s knowledge is lacking [20] which may continue into adulthood [21]. In the present study in regard to dairy products children’s emerging themes displayed an incomplete understanding, e.g.: 

“It can kill you if you have too much dairy”

Dietary reference values for fat intake for children aged 10–11 in the UK are 35% of total energy intake [22] with a recommendation to consume some milk and dairy products. This has implications for intervention design and is suggestive of the need for clear information about how to adopt a healthy diet and *translate* knowledge into practice, rather than a sole focuss on the benefits or constituents of a healthy diet through typical educative based means.

The important role parents play in children’s eating behaviours and food intake was evident from the children and adults’ pen profiles, both in terms of barriers to, and the child as a change-agent for healthy eating. The role of parent’s contributions to children’s eating behavior has been noted previously as multifaceted and complex [23,24]; but can be separated into overt and covert control [25,26]. Overt control includes monitoring and regulating children’s eating behavior, and was evident in this study, for example, children not being allowed to leave the table before eating vegetables, and through restriction of dietary fat and chocolate (Figure 3). This overt control can be counterproductive leading to increased portion sizes [27], dietary restraint and disinhibition [28,29], and is implicated in overeating and overweight [30,31]. However, overt control also has a positive relationship with healthy snacking, fruit and vegetable intake, and reduced intake of energy dense foods [26]; [25]; [32]. Examples of covert control emerging from the focus groups included reducing access to sweets in the home (Figure 4), a practice associated with a decrease in unhealthy snacking [26] and an increase in FV intake [25]. Parental control practices therefore can influence both positive and negative eating behavior but more research is needed in this area to fully understand the complex relationships between families and eating behavior.

The pen profiles also revealed parental support and encouragement of healthy behavior by educating children to make healthy choices whilst shopping, enhancing choice through tasting FV, and encouraging interaction with health education at schools (Figure 4). Family support can protect adolescents against unhealthy choices [33] and parents have been shown to be supportive of interventions on health and well-being at schools [34]. Pen profiles suggest schools are providing (some) health education and parents are generally supportive : 

"Child: “And they encourage like in school they encourage us to eat healthy stuff”."

"Adult: “They had a well-being day at school. They had loads of stalls and different organizations in. They do try to educate people”"

The interaction of parents, children, schools and health is a complex issue and research in this area remains in its infancy. However, previous research [35] has shown that 9–10 year old school children are receptive to interventions and small behavior changes and motivational practices for families may be possible. Indeed, Watson et al., [36] showed improvements in BMI in children were related to adult changes in BMI suggesting a strong interaction of family behavior. Future interventions should investigate these interactions in more detail and how they may shape the future well-being of children. Clearly, an intervention targeting improvements in diet must include some targeted family or parental component, and raises the possibility of family orientated home-school link tasks or parental engagement sessions as possible mechanisms to positively influence the parental role upon food intake.

The emerging themes relating to barriers to healthy eating showed that external drivers such as advertising, the preferred sensory experience of “unhealthy” foods, and food being used as a reward may play a role in preventing healthy eating, in particular FV consumption (Figures 5 &6). The sensory experience of FV consumption, including taste, smell and appearance, has shown a consistent relationship with fruit intake with the taste of vegetables presenting the major barrier [20,37]. Krølner et al. [20] highlighted that the sensory experience can influence willingness to consume FV; with vegetables described as bitter, and the taste of unhealthy food preferred. The present study data are in agreement with children preferring the taste and visual experience of chocolate, pizza, and chips (Figures 5 &6), which is in line with previous evidence that suggests children prefer the taste of unhealthy foods over healthy foods. [38] The sensory experience of FV has been shown to be related to children’s sensitivity to taste and smell and more gradual approaches to introduce FV into the diet have been suggested [39]. Furthermore, introducing a variety of healthful foods and exposure can encourage greater intake [20] however, repeated attempts by parents to encourage intake of specific foods can lead to frustration and parents may eventually may stop trying [40] and thus limiting children’s choice of FV.

The practice of showing children pictures of FV has been shown to increase intake and variety of fruit, however this had no effect on vegetable intake [41]. This suggests visual exposure may be a beneficial potential strategy for children with respect to fruit but not vegetables. However, sensory based nutrition interventions are still in their infancy [37] and further scientific evidence with well-designed studies with an emphasis on longer term monitoring are warranted.

Product marketing, via the TV in particular, was highlighted by both adults and children in the present study (Figures 5 &6) with parents showing concern with their children’s desire for “rubbish food”. TV viewing has been shown to positively correlate with BMI in children [42] and that children watching adverts relating to “junk food” had a more positive attitude towards this type of food [43,44].

Other barriers to healthy eating were identified through themes linked with convenience with children stating:

*“I just eat anything that is in the fridge”* (Figure 5)

While parents’ themes were based on money, time, and tiredness (Figure 6, N.B. the term ‘shattered’ refers to tiredness). A previous study of mothers’ perceptions with respect to healthy eating showed that key themes were time, money and convenience were reasons for not eating healthy. Specifically, mothers were aware of public health messages on healthy eating however they were not confident of making changes to improve diet [45]. Future interventions should respect the limitations of household finances and perceived time constraints that may in turn prevent adoption of a healthy lifestyle of parents, and encourage motivation in parents through involvement.

A number of strengths are apparent in the present study. Firstly, by including participants from varied socio-economic backgrounds, known to be important in health-related behaviours, the findings may be applied across socio-economic groups. Furthermore, the relatively large sample size, whilst using the pen-profiling technique further advances the literature using this methodology. Triangulating data between parents, children and teachers decreased the risk of misinterpretation of data, and also improves the credibility, dependability and transferability of the findings. Furthermore, the key messages from this study will be used to inform and shape the healthy eating component of the CHANGE! intervention, ensuring the intervention is specific to the target population.

In terms of limitations, participation bias may have impacted upon results, with 37% of children invited to take part refusing to participate, despite this the majority of those invited consented to participate and represented a range of socio-economic backgrounds.

## Conclusions

The study findings indicate that children and adults had generally sound knowledge of the constituents of a balanced diet. Data suggest that knowledge related to diet composition was not a barrier per se to healthy eating within the population group studied and education showing how to translate knowledge into behavior or action is required. It was evident that parents provided support and encouragement of healthy behavior by educating children to make healthy choices and enhancing choice. The pen profiles also revealed the role children can have as change-agents for healthy eating at home. The emerging themes showed that external drivers such as advertising, the preferred sensory experience of “unhealthy” foods, and food being used as a reward may play a role in preventing healthy eating. The key themes that emerged through this study will be used to inform and tailor the healthy eating component of the CHANGE! intervention study.

## Competing interests

The authors can confirm that there are no known competing interests for the present study.

## Authors’ contributions

LMB drafted and edited the manuscript and contributed to the design of the CHANGE! project. ZRK gave expert input on the pen-profiling technique drafted and edited the manuscript IGD gave expert nutritional input, drafted and edited the manuscript LH conducted pen-profiling analysis GW and KM conducted the focus group interviews and contributed to the construction of the manuscript SJF drafted and edited the manuscript and is the principal investigator for the CHANGE! programme of research. All authors read and approved the final manuscript.

## Pre-publication history

The pre-publication history for this paper can be accessed here:

http://www.biomedcentral.com/1471-2458/12/710/prepub

## Author details

^1^The Research Institute for Sport and Exercise Sciences, Liverpool John Moores University, Tom Reilly Building, Byrom Street, Liverpool L3 3AF, UK.^2^The Faculty of Education, Community and Leisure, Liverpool John Moores University, IM Marsh Campus, Barkhill Road, Liverpool L17 6BD, UK.
